# 

**Published:** 1906-09-15

**Authors:** 


CONTENTS.

**School Diseases—Preventable, but not Prevented** 415
**Professor Behring's "Tulase"—New Remedy for Consumption** 416
**Annotations** 417
**Medical Opinion and Movement** 418
**Graduate Teaching Facilities in London Hospitals** 419
**Hospital Clinics—**
Intestinal Parasites 422
**Progress in Medicine and Surgery—**
Notes on Liquid Articles of Dietary 422
**Practical Notes on Diagnosis and Treatment** 424
**Special Analytical and Health Commission on Tinned and Preserved Food** 425
**Bakers' Exhibition** 426
**Remedies and their Uses** 427
**New Appliances and Things Medical** 427
**Hospital Administration—**
New Workhouse Infirmary at Stepping Hill, Stockport 428
The Relation of Insanity to Civilisation.—II. 431
The Story of the Insane from Year to Year 432

*NURSING SECTION.*

**Notes on News from the Nursing World—**
An Indictment of the Midwives Act—The Perils of Nursing Classes—Dismissal of a Superintendent Nurse—Candidates for a Cottage Hospital Matronship—What is a Probationer?—A Compromise at Lynn—Army Sisters in Camp—The Training Schools Recognised by the Midwives Board—Pianos for Poor-law Nurses—An Appeal from Western China—Nursing Association or Cottage Hospital?—Amateur Cooks—Death of a Matron—A Protest at Middlesbrough—Fever Hospitals and the Bible—Admirable Progress at Darlaston—Raising the Wind at Crediton—Short Items 337-340
**The Nursing Outlook** 341
**The Care and Nursing of the Insane** 342
**The Nurses' Clinic** 343
**Incidents in a Nurse's Life** 344
**A Week's Experience in a Canadian Hospital** 345
**A Floral Fancy** 345
**Appointments** 346
**Everybody's Opinion** 347
**A Book and Its Story** 348
**Notes and Queries** 349
**Notes from India** 350
**For Reading to the Sick** 350

